# Ateriovenous subclavia-shunt for head and neck reconstruction

**DOI:** 10.1186/1746-160X-4-27

**Published:** 2008-11-24

**Authors:** Rita A Depprich, Christian D Naujoks, Ulrich Meyer, Norbert R Kübler, Jörg G Handschel

**Affiliations:** 1 Heinrich-Heine-University Duesseldorf, Department of Cranio- and Maxillofacial Surgery, Moorenstr. 5, 40225 Duesseldorf, Germany

## Abstract

Reconstruction of the facial hard- and soft tissues is of special concern for the rehabilitation of patients especially after ablative tumor surgery has been performed. Impaired soft and hard tissue conditions as a sequelae of extensive surgical resection and/or radiotherapy may impede common reconstruction methodes. Even free flaps may not be used without interposition of a vein graft as recipient vessels are not available as a consequence of radical neck dissection.

We describe the reconstruction of the facial hard- and soft tissues with a free parasacpular flap in a patient who had received ablative tumor surgery and radical cervical lymphadenectomy as a treatment regimen for squamous cell carcinoma (SCC). To replace the missing cervical blood vessels an arteriovenous subclavia-shunt using a saphena magna graft was created. Microvascular free flap transfer was performed as a 2-stage procedure two weeks after the shunt operation. The microvascular reconstructive technique is described in detail.

## Background

Various reconstructive options have been used in the past for the reconstruction of head and neck tissues. In a high number of patients, mainly treated because of head and neck carcinoma, tissue defects may develop following tumor therapy because of extensive resection or chronic effects of radiotherapy. In the initial years, reconstruction was limited to the use of pedicled flaps such as the pectoralis major, latissimus dorsi, and deltopectoral flap [1-3]. Introduction of free flap surgery in the 1970s due to technological advances in microsurgery led to significant refinements of reconstruction techniques [4-6]. Although most cases can be managed with free flaps some patients present with unavailable blood vessels due to the consequences of radical cervical lymphadenectomy [7]. The absence of anastomotic sites, especially venous recipient sites, obviate reconstruction of the maxillofacial region by a 1-stage microvascular reconstruction technique. For these challenging cases construction of an arteriovenous fistula as alternative arteriovenous recipient site is necessary before free tissue transfer is indicated.

This report describes the use of a vena saphena magna interposition ateriovenous subclavia-shunt for the reconstruction of complex defect in the head and neck region.

## Case presentation

**Figure 1:**
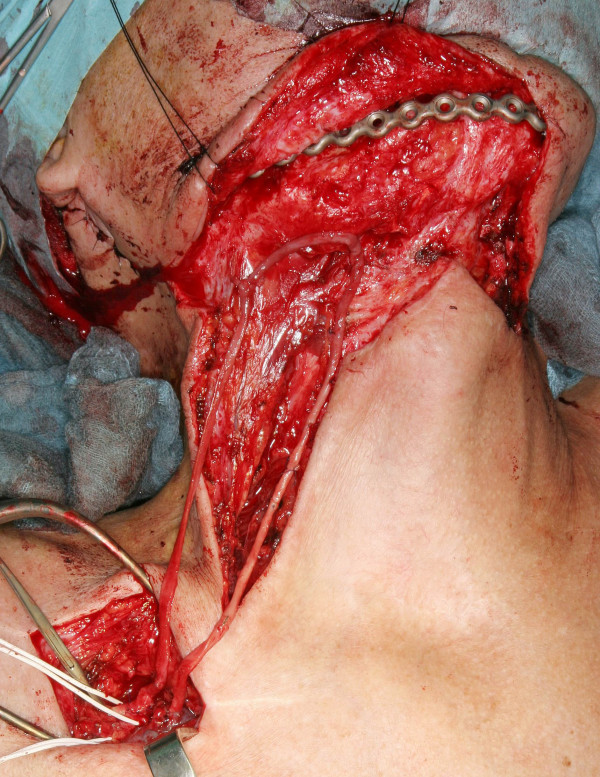
**Surgical approach to the subclavian vessels**. The saphena magna vein is end-to-side anastomosed to the subclavian vessels. The length of the vessel loop matches the length of the free flap's pedicle.

**Figure 2:**
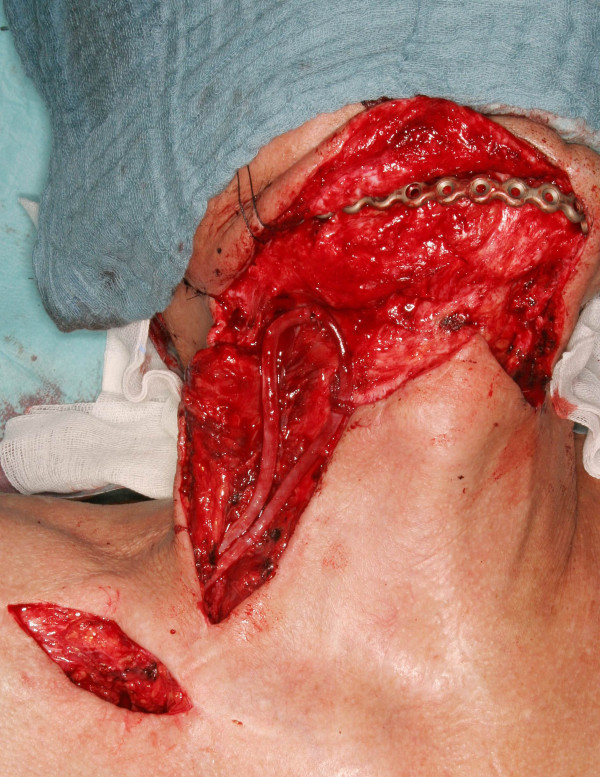
Arteriovenous loop pulled through the supraclavicular skin bridge and fixed in the desired region.

**Figure 3:**
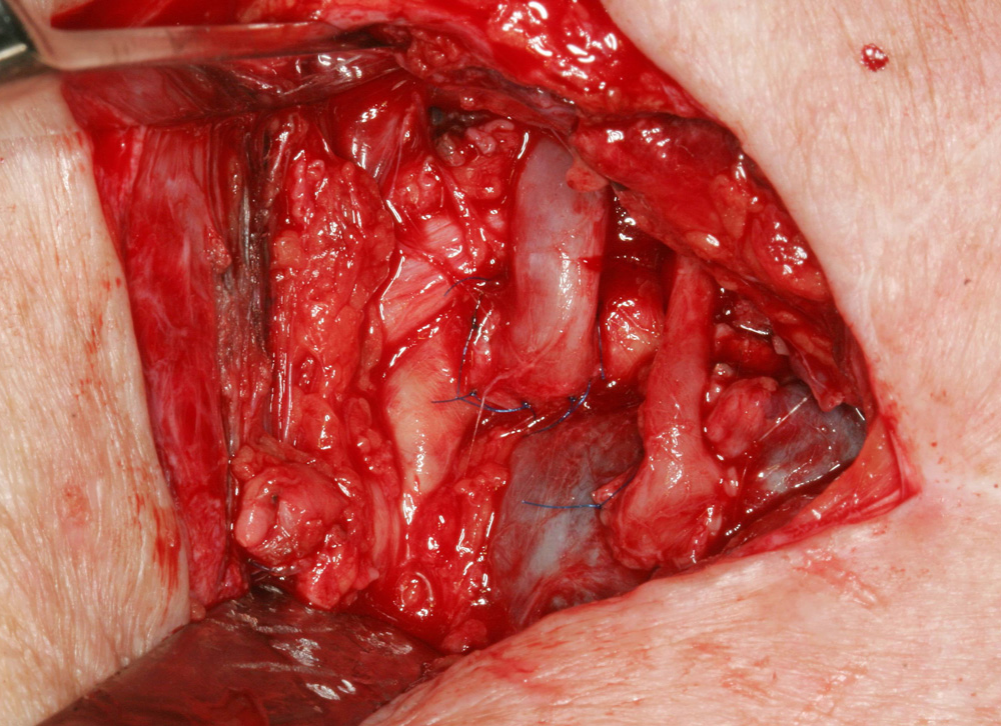
End-to-side anastomoses to the subclavian vessels, suture material 8-0 Ethilon (Ethicon, Norderstedt Germany) (artery left, vein right).

**Figure 4:**
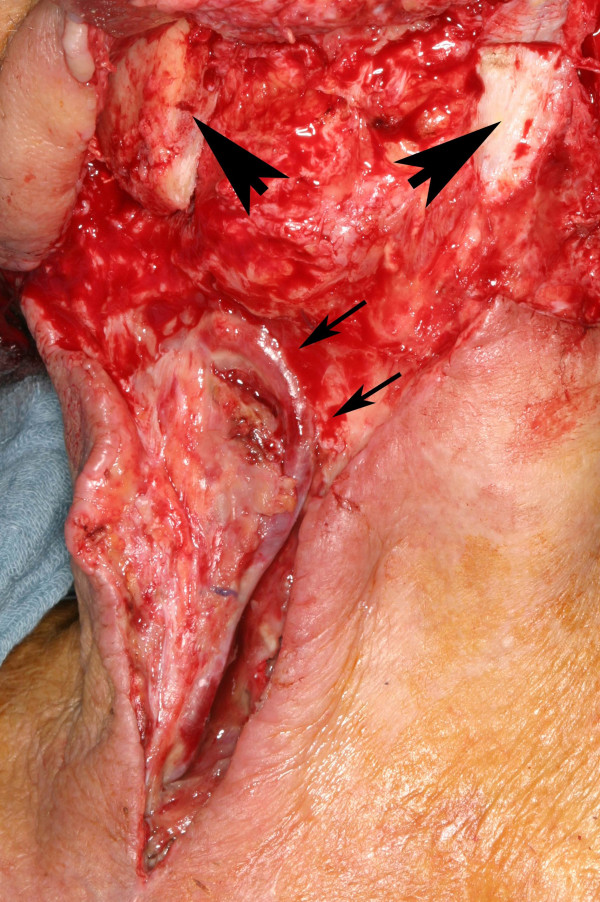
**Exposition of the vessel loop after 10 days of healing**. The vessel wall is thickend, no signs of inflammation (small arrow) are present. Large arrows indicate the resected mandibular stumps.

**Figure 5:**
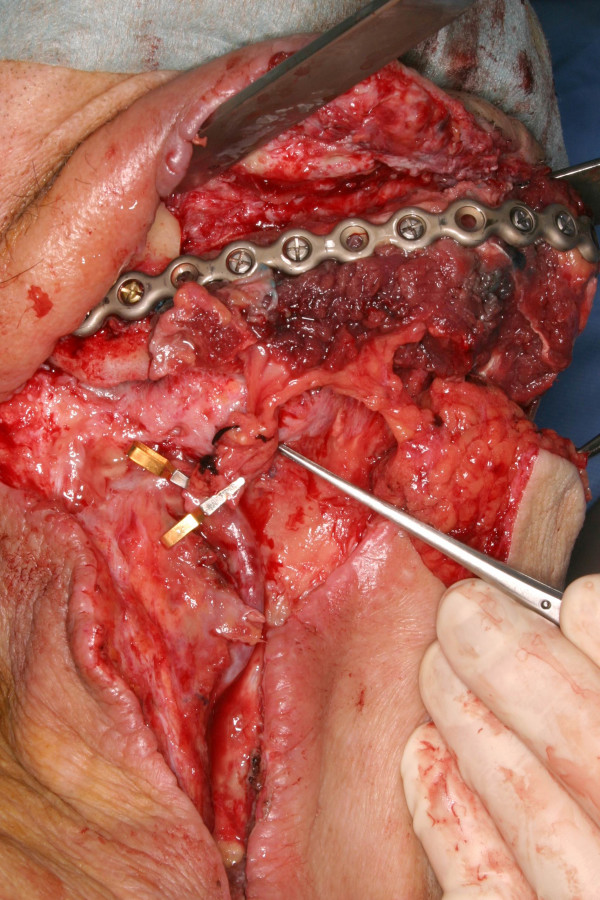
**Sufficient length of the pedicle of the scapular flap (tip of the forceps: middle of the free flaps pedicle, goldclips: anastomotic sites of vessel loop)**. The microvascular anastomosis can be performed without tension on the vessels.

In a 61-year-old male with a history of alcohol and nicotin abuse but no other serious diseases the initial diagnosis of a SCC of the right anterior floor of the mouth and cervical lymph node metastases (pT3, pN2b, pM0, G2) was made. He underwent former surgery including partial resection of the tongue, the mandible and of the floor of the mouth, radical neck dissection of thr right side, selective lymphadenectomy on the left side, immediate reconstruction of the mandible with a reconstruction plate, and intraoral reconstruction with a pectoralis major flap and irradiation post surgery (60 Gy). As a consequence of woundhealing based on the postoperative radiotherapy a large extraoral soft tissue defect and exposition of the reconstruction plate occurred. In order to reconstruct the soft tissues and to establish conditions for a secondary bony defect reconstruction a vessel reconstruction procedure was performed prior to free flap surgery. The saphena magna vein was taken from the patient's right leg. One end of the vein was anastomosed in an end-to-side fashion to the right subclavia artery and the other end in an end-to-side fashion the to the right subclavia vein thus resulting in an arteriovenous loop (fig. 1, 2, 3, 4). After 10 days of healing microvascular free tissue transfer of a osteomyocutan parascapular flap was perfomed. The vessel loop was divided and an end-to-end anastomosis of the flap artery to the arterious branch of the shunt was performed (fig. 5). The flap's vein was anastomosed to the venous branch accordingly. Healing of the transplant was uneventful. No donor-site deficits occured following parascapular flap harvest. Postoperative radiographic and scintigraphic examination showed successful bridging of the bony defect and successfull integration of the osteomyocutan parascapular flap.

## Discussion

Since the introduction of a microsurgical free flap for the reconstruction of a head and neck defect in 1976 a wide variety of free flaps have been described for the reconstruction of the head and neck region [8]. As since then many different free flaps have been shown to provide excellent reconstructive results free microvascular tissue transfer has become a popular method for repair of large head and neck defects [4,9]. Due to the robust blood supply free flaps improve outcomes of healing even under impaired soft and hard tissue conditions although free flap survival rate in secondary reconstruction is significantly lower than that in immediate reconstruction because of the limitation of available recipient vessels, significant scar formation and delayed wound healing due to irradiation [10].

In situations when microvascular free flap transfer is impeded as recipient vessels are lacking because of surgical ablation or radiation damage pedicled could be used [11,12] or remote vascular access could be required [13]. As these alternative vessels are often at a greater distance from the primary defect the length of the vascular pedicle is becoming a limiting factor. Options to increase the length of the vascular pedicle include selection of an alternative flap with a longer vascular pedicle, use of an interposition vein graft, or creation of an arteriovenous loop [14]. Since the first publication in 1982 by Threlfal et al. several authors described the use of temporary arteriovenous fistula and microsurgical free tissue transfer for the reconstruction of difficult defects in all body regions [15,16]. The technique can be used as a 1-stage or 2-stage procedure which technique is superior still remains controversal [16].

In the head and neck region recipient vessels are predominantly branches of the external carotid artery and jugular vein [13,14]. Contralateral neck and face vessels are used when potential recipient vessels on the side of the defect are compromised [17,18]. Some authors recommend direct anastomosis to the carotid artery or the jugular vein [19]. A different technique is the use of a transposed cephalic vein in cases when the recipient local vein is lacking [20].

Our patient had undergone previous tumor resection and soft tissue reconstruction, radical neck dissection and post-operative radiation. The usual options for (contralateral) recipient vessels i.e. branches of the external carotid artery and jugular vein were not available as was revealed by pre-operative angiographic examination. The creation of a saphenous arteriovenous loop using the subclavia artery and vein offered a reliable and practical alternative reconstruction method when no pedicled flap or other recipient vessels were available. The ateriovenous subclavia-shunt for such difficult cases seems to be a reliable alternative because of its advantages in the following aspects: 1. The subclavian vessels are located outside ablative surgical field, zone of radiation or injury. 2. The vessels' size is suitable for microsurgical anastomosis, especially the vein can accept high venous outflow from the graft. 3. Anatomy of the subclavian vessels is constant and offers favorable surgical approach. 4. The two stage approach with a 10- to 14-day interval between loop construction and free tissue transfer allows the vein to thick the vessel wall and to avoid venous collapse even in cases of low venous flow.

## Consent

Written informed consent was obtained from the patient for publication of this case report and accompanying images. A copy of the written consent is available for review by the corresponding author.

## Competing interests

The authors declare that they have no competing interests.

## Authors' contributions

RAD drafted the manuscript and performed the operation. CN drafted the manuscript and performed the operation. UM participated in the planning of the operation and performed it. NRK participated in the planning of the operation and performed it. JGH participated in the planning of the operation and performed it. All authors read and approved the final manuscript.
